# Carbon Footprint of a Port Infrastructure from a Life Cycle Approach

**DOI:** 10.3390/ijerph17207414

**Published:** 2020-10-12

**Authors:** Rodrigo Saravia de los Reyes, Gonzalo Fernández-Sánchez, María Dolores Esteban, Raúl Rubén Rodríguez

**Affiliations:** Department of Civil Engineering, Universidad Europea de Madrid, 28670 Villaviciosa de Odón, Madrid, Spain; rsaraviadelosreyes@gmail.com (R.S.d.l.R.); gonzalo.fernandez@ext.universidadeuropea.es (G.F.-S.); raulruben.rodriguez@universidadeuropea.es (R.R.R.)

**Keywords:** greenhouse gases emissions, port infrastructure, carbon footprint, life cycle assessment

## Abstract

One of the most important consequences caused by the constant development of human activity is the uncontrolled generation of greenhouse gases (GHG). The main gases (CO_2_, CH_4_, and N_2_O) are illustrated by the carbon footprint. To determine the impact of port infrastructures, a Life Cycle Assessment approach is applied that considers construction and maintenance. A case study of a port infrastructure in Spain is analyzed. Main results reflect the continuous emission of GHG throughout the useful life of the infrastructure (25 years). Both machinery (85%) and materials (15%) are key elements influencing the obtained results (117,000 Tm CO_2_e).

## 1. Introduction

In the world, there is a high demand for achieving a balance between daily activities and natural resources [1]. The concept of sustainability aims to improve environmental conditions by promoting the balance between human development, the surrounding environment, and the resources. Although it is essential to recognize that the sustainability trend has improved, there is still a lot of work ahead, and the development of new methodologies [2,3] that encompass the development of innovative techniques is necessary, as well as tools that analyze and evaluate the present and future state of natural resources.

Human activities are measured in economic and social costs. However, there is a measurement [4] that is often overlooked, perhaps as it is not directly detected, which is the environmental cost. Human development generates an environmental cost that is represented by greenhouse gases (GHG). Recent trends position GHG as one of the main aspects to be mitigated by the extensive and continuous emissions generated [5].

GHG are made up of different gases, with carbon dioxide (CO_2_), methane (CH_4_) and nitrous oxide (N_2_O) being the most important ones. The factors that influence radiative forcing are the following: Radiation power, and average time the gas molecule remains in the atmosphere [6]. These two factors make up the global warming potential (GWP) of each gas, whose unit is the kilogram of equivalent CO_2_. In other words, each gas analyzed is transformed to kg of CO_2_ units using an equation sanctioned by practice.

The concentration of atmospheric CO_2_ exceeded 417 parts per million (PPM) in 2019, a fact that sets a historical record. The observatory that carried out that research was Mauna Loa in Hawaii (National Oceanic and Atmospheric Administration—NOAA) [7]. From the statistics provided by NOAA, it may be concluded that the CO_2_ emission trend is exponential and that these results are exceeded each year (Table 1).

The cause of the uncontrolled growth rests unquestionably on human activity. The burning of fossil fuels for energy production, transport, and industry has increased in recent years [8,9]. In the unexpected situation such as the pandemic caused by COVID-19, the impact of anthropogenic activity was observed, through a weekly reduction in greenhouse gases of 30% during the period of confinement [7].

Until present, the procedure that is most effective in determining the levels of emissions generated is measuring the carbon footprint (CF) [10]. This concept quantifies the carbon dioxide (CO_2_) emissions generated, produced directly and/or indirectly by an activity, or by the set of activities during the life cycle of a product [11]. One of the most important advantages of the CF calculation is that it is possible to adapt it to any project [12]. Therefore, all sectors should be obliged to perform CF calculations in the near future.

The above should even be made more so in the construction sector, which has a significant impact on the environment due to large earth movements, the treatment of compound materials, and land modification [5]. Currently, the construction sector is responsible for 20% of the emissions generated worldwide, being the first in materials consumption [13].

Life Cycle Analysis (LCA) is a methodology that allows the calculation, evaluation, and interpretation of the generated emissions during the life time of an infrastructure, thereby showing the GHG produced during all the project phases [14,15].

In the civil engineering sector, seaports are the infrastructures where the generation of GHG is highest. On the other hand, ports are considered dynamic centers within the international market where numerous services and benefits are integrated [16,17]. It should be noted that, although the literature in relation to these infrastructures is not very abundant, it has been shown that the greatest impact is produced by large machinery and ships than by the construction of infrastructure. Some references showing the previous statements include: A study on the annual climate change and primary use of Swedish transport infrastructure, including roads, railways, airports, and fairway channels [18]; evaluation of the latest trends in the cargo handling equipment industry in ports [19]; the life-cycle emissions from port electrification in the case study of the Port of Los Angeles [20].

In the absence of research, and therefore of precise conclusions that accurately describe the reality of these infrastructures, a study was carried out that focused on the Life Cycle Analysis of a port, which considered both the phases of construction and operation. This manuscript summarizes this research. The selected project was carried out in Spain, and the infrastructure considered in the research consists of the construction and maintenance of a quay of approximately 32,000 m^2^, being characterized by the use of caissons.

On the other hand, it is important to consider that most of the cases in which this methodology has been applied are outside the scope of the civil engineering sector [5,21,22,23], and it is, therefore, a challenge to apply it to port infrastructures. However, there are very specific cases that illustrate the large emissions that are generated in this sector.

Previous research typically focused on the individual performance of materials [24,25] and the behavior of different materials in the overall infrastructure [26,27,28,29,30]. GHG research in maritime engineering is scarce, which is the key aspect to understand the importance of this research. However, it has been essential to consider the approaches developed in this sector [31,32,33] to carry out this study.

## 2. Objectives

This study mainly seeks to know the emission ranges produced during the entire operational life of the selected infrastructure. This involves analyzing all phases (construction and maintenance), as well as all the elements (materials and machinery). This approach permits the characterization of the problem of greenhouse gases in port infrastructures, an aspect that has never been studied up until now. The main objectives of this research are:

- Characterization of the GHG problem in maritime infrastructures.
- Calculation of the emissions produced in a maritime project that includes both construction and maintenance.
- Identification of aspects with the highest GHG emissions.

## 3. Scope

The scope established in this research corresponds to the Life Cycle Analysis of a port construction. It is important to mention that both the construction and maintenance have been considered. Although each construction is unique, various studies related to roads [13,27,28,29,34], waste management [22], or the specific elaboration of materials [24,26] have been taken into account to build a powerful methodology that begins to illustrate the problem of GHG emissions in ports. The whole of the infrastructure life cycle has been considered, which includes materials extraction, production, transportation, port construction, and maintenance (operation and conservation), but not the port dismantling. The transport and energy upstream chain was also considered.

**Figure 1:**
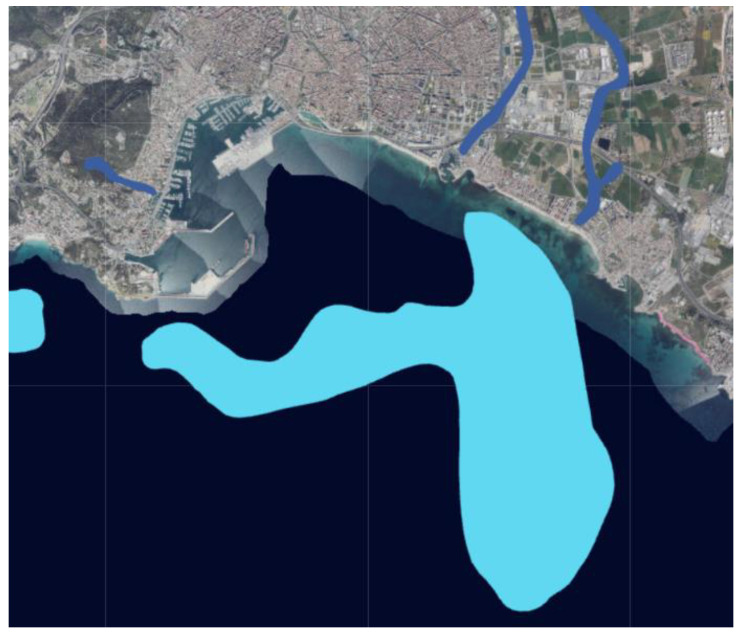
Posidonia meadow (in blue color) close to the construction area.

The elements included in this LCA can initially be classified into three groups: Machinery, materials, and natural systems. As there are still no references that reflect a clear and concise calculation for the seabed, this element has not been considered in this research. Considering natural systems, Posidonia Oceanica is an algae considered as protected species in the Mediterranean Sea (Figure 1) [35]; laws establish a prohibition regarding the disruption of that species, which are so important in the absorption of CO_2_ [36,37,38].

In relation to the machinery, it was decided to analyze only the use of the machines during construction and maintenance. That means that the manufacturing of the machinery itself is out of the scope of this research, an aspect in which emissions are also generated.

Table 2 summarizes the most important information and LCA concepts included in this research for studying the GHG emissions in construction and maintenance of a port infrastructure [15].

## 4. Methodology

The methodology that has been used for the management information data is based on CO2NSTRUCT [14], which has an in-depth database containing more than 300 data elements (materials, off-road machinery, transport machinery, electricity mixes, energy sources, and types of waste), and which includes European and Spanish data.

The materials use a three-step approach following the CO2NSTRUCT approach: Pre-production and production (related to construction stage), and installed material (related to maintenance stage).

- The pre-production emission is related to energy consumption, process emissions, and transportation to final factory (e.g., the machinery used for the extraction of raw material, transport machinery to the processing facilities) [29].
- Production is based on the emissions generated for the final processing of materials. In this case, the emissions make reference to the final product established in the work units necessary for the construction of the work [29].
- Installed materials make reference to artificial surfaces created after the construction that can be carbonated during the life cycle such as concrete that can absorb up to 3800 kg CO_2_/m^3^ in a period of 100 years according to Galan et al. [39].

On-site activities focus on the construction of the port infrastructure, as well as the maintenance activities. The machinery is classified into two groups:

- Transport machinery: Machinery used to transport the material to the site [40,41]. EMEP/Copert methodology tier 3 was used.
- Construction machinery or off-road machinery: Specific machinery to carry out the activity established in the work units, involved in the construction activities [40,41]. It used EMEP methodology tier 3.

As the construction scope is well-defined in the as-built project, in this research, a maintenance plan for a port was presented considering all the characteristics of the specific project and location conditions. Although the maintenance plan is based on estimations from good lessons and experts, the calculation is done in a similar way as in the construction stage.

Waste management (related to energy consumption in its treatment and transportation from site) and environmental systems were also initially considered [28]. Energy consumption, from the so-called pre-combustion (source production and distribution in electricity or fuel production and distribution), and its combustion with national emission factors allow us to obtain the data for energy emissions related to materials, machinery, and transportation. For more information about this methodology, the in-depth detailed methodology from [14] was followed. Emission factors of the electrical mix, materials, and fuel combustion of the off-road machinery were updated with the last published data following national and European reports (EMEP/EEA air pollutant emission inventory) from the system and database referenced.

## 5. Study Case

### 5.1. Construction Stage

The case study selected was located in Spain. The quay construction began in late 2017, with the project lasting a duration of 37 months, and the total cost of the project was around 35 million Euros. More than 150 off-road machinery were identified on the as-built construction project. The main activities of the port expansion considered for this case study were:

- Dock extension: 215 + 130 + 24 m, with submerged concrete blocks for passenger traffic. Eleven floating drawers of around 34 m length with granular filling cemented on breakwater bench, with a superstructure including bollards and fenders.
- Enlargement of facing to 100 m, also with a dock with submerged concrete blocks.
- Harbor dike extension: 115 + 410 m, with concrete drawers in breakwater benches.
- Esplanade: Reinforced concrete plates with tongue and groove modular elements. A bituminous mix finish.

The most important materials in the construction stage are concrete and granular materials, with some important activities such as the esplanade and pavement of the dike and dock. Most of sand and gravel came from dredging. This recycling strategy will reduce the GHG footprint by reusing this kind of material used to fulfill the drawers. Off-road machinery age is considered on an average of around 4 years. Natural systems were initially considered but most of the parts were directly above the sea, so just a small impact on land-use and change in land-use is included.

### 5.2. Maintenance Stage

**Figure 2:**
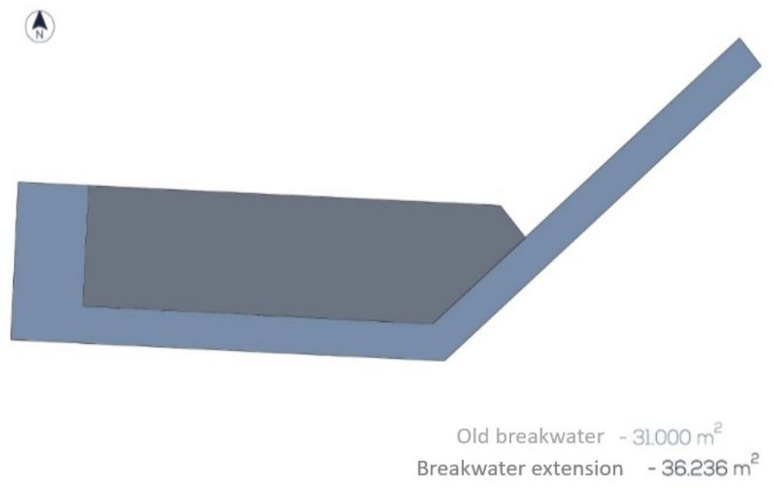
Distribution of the maintenance area to carry out the relevant work.

A maintenance and conservation plan for the port was defined. Using existing guides and manuals [42,43,44], and our own experience, a maintenance plan was designed tailored to project conditions (Figure 2). The type and number of inspections were detailed in the maintenance plan. The actions to be carried out are listed in Table 3, including the following information in each operation: Area where the task is going to be carried out, main material, surface, thickness and volume affected, frequency of carrying out the task and percentage affected, number of operations during the life time of the infrastructure, ratio of operations in the life time, and ratio of material volume divided by the frequency.

## 6. Results

### 6.1. General Results

**Figure 3:**
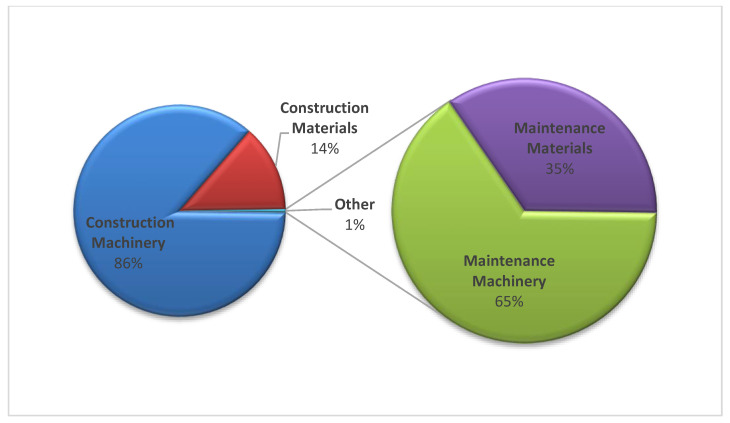
Distribution of the phases analyzed in this research: Results in percentage (%).

The results obtained in this research are aligned with the expected trend included in [29] (see Figure 3). In fact, Figure 3 shows that main emissions occur during the construction phase (close to the 100% of the total emissions), with the emissions from the operation and maintenance phases making up only 1% of the total emissions. Furthermore, the proportion of CO_2_ emissions is the greatest, leaving the other GHG with almost zero proportions in total. The result of Tm of CO_2_e for the entire project is 117,000 Tm, that is, only taking into account the construction and maintenance phases.

Machinery, and more specifically, the construction or off-road machinery, is the most influential element. On the contrary, the machinery used for the transport is the minority component. In addition, 14% of emissions correspond to materials during the construction phase. Within this component, pre-production is the phase with the greatest impact with almost 90% of total CO_2_ material emissions.

The activity in the construction stage, which is the highest contributor to the GHG, is the construction of the Esplanade. The influence of the off-road machinery (Table 4) is such that it makes this activity stand out.

In the construction stage, it is clearly observed that the off-road machinery is the element that generates the most emissions. Transportation is three orders of magnitude lower. Table 5 shows the breakdown of the results obtained at the construction stage.

As maintenance work is important and almost nullified by the importance of construction, this section is established to interpret the values obtained during this phase. The general trend, although with lower values, is the same as in the construction phase where the off-road machinery generates almost all emissions. It is worth mentioning that the distribution between the elements is a little more balanced. Table 6 breaks down the results obtained for the maintenance stage of off-road machinery, transportation, and materials.

### 6.2. Scenarios

From the case study and obtained results, some sensitivity analysis was made according to the detail of data used in the study. Following some scenarios created by [45] from road projects, four scenarios were studied in depth to illustrate the emission ranges of a maritime construction and to observe the sensitivity of the proposal and results. The four scenarios used were:

- Scenario 1 is based on variables depending on off-road machinery as it is the most important element. The age of the off-road machinery has been changed from 4 years from the base case to 20 years.
- Scenario 2, following the previous case, is that the technology of the off-road machinery has been modified, and so has the transport machinery, to the most actual one from that which was in the construction project to check the sensitivity to this variable.
- Scenario 3, going in depth in the machinery element, is that biodiesel fuel is established instead of diesel. Although the performance can be lower, the emissions in the case of using biodiesel B20 fuel values are 20% less than in the case of using diesel fuel.
- Scenario 4 is focused on materials. This is because machinery is considered “well-calculated” as in most actual European guidelines, tier 3 is used. But what about materials? We have used national data, but it is not the actual exact information from the construction of the port infrastructure. To measure the sensitivity to this element, a known database has been used (ICE, the Inventory of Carbon Energy [34]) for measuring the sensitivity of global results changing the raw data of elements used.

**Figure 4:**
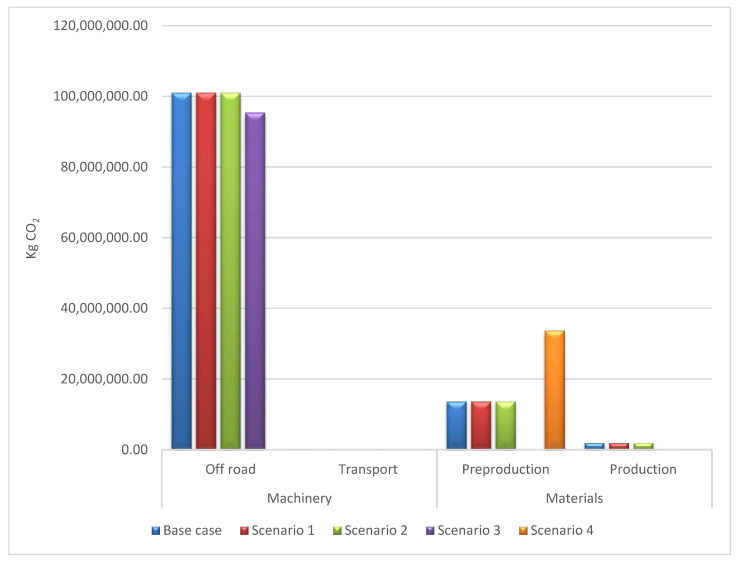
Results of CO_2_ kg emissions obtained in each scenario: Construction phase.

The scenarios served to obtain a broader vision of the importance of the factors that participate in this process. As can be seen in Table 7 and in Figure 4, large changes are only detected in scenario 3 and in scenario 4.

**Figure 5:**
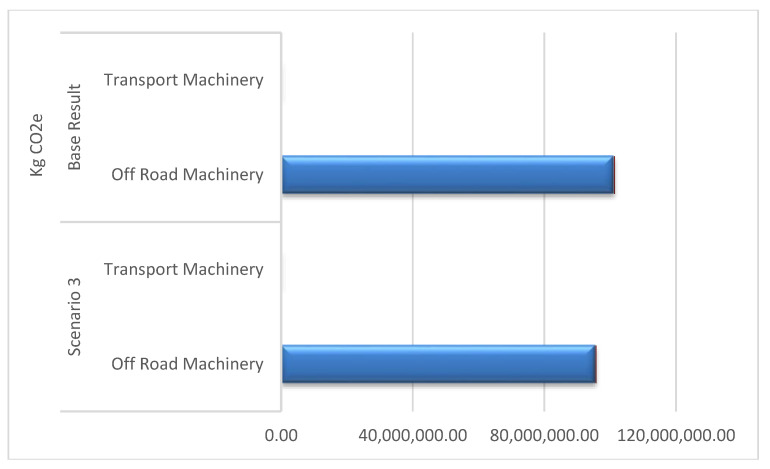
Results of kg CO_2_e emissions obtained in scenario 3 compared to the base result.

Scenario 1 and 2 are considered near to the base case results. New technologies are more focused on reducing NO_x_ and PPM than on CO_2_, and age is not a main factor when the initial average is 4 years. Scenario 3 shows important changes that can open new work scenarios focused on renewable energies. The use of biodiesel as fuel reduces emissions by approximately 7% in the construction phase and 8% in the maintenance phase. The results can be viewed in Figure 5.

**Figure 6:**
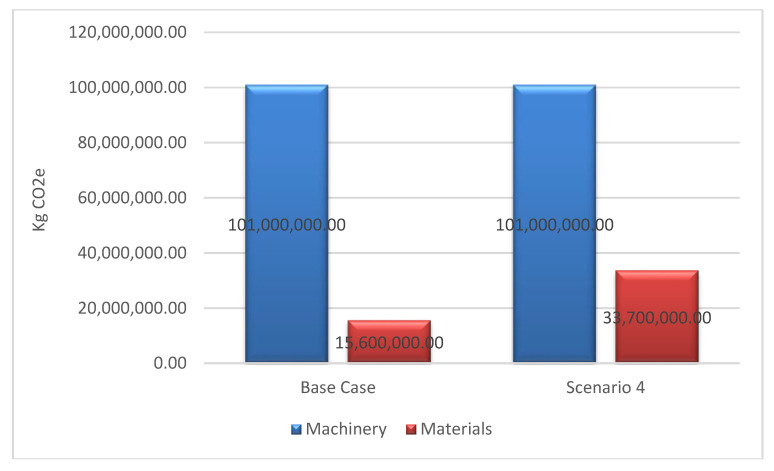
Results of CO_2_e kg emissions obtained in scenario 4 compared to the base result.

In the case of scenario 4, the information database changes completely. The Inventory of Carbon Energy [34] database is used, which uses a more general methodology as it treats pre-production and production as the same stage (Figure 6).

**Figure 7:**
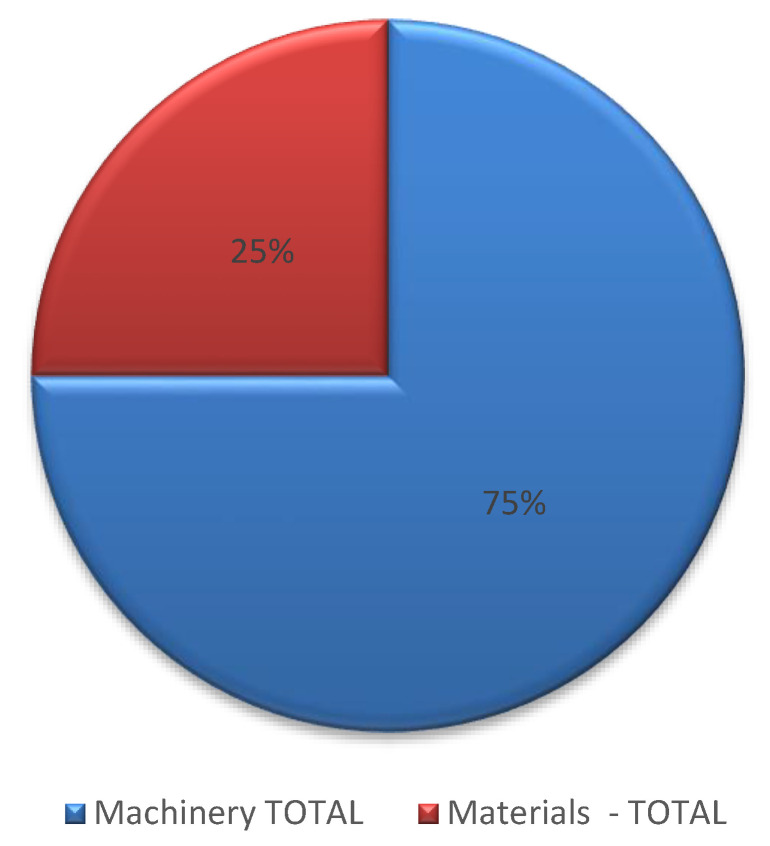
Total greenhouse gases (GHG) distribution in scenario 4.

It must be remembered that materials only account for about 14% of all project emissions. In this scenario, 33,700 Tm CO_2_e is obtained (Figure 6), which means that the materials increase 216% (Table 7). Therefore, the total distribution in this scenario is more balanced, as can be seen in Figure 7, which shows that the distribution of emissions in scenario 4 is around 75% due to machinery, and 25% due to materials.

## 7. Discussion

This research begins to reflect the large volumes of GHG emissions produced in the construction sector. Although there are references that analyze this phenomenon in other areas of the construction sector, this document is the pioneer in evaluating and interpreting the GHG emissions in maritime construction. Although various references were consulted [13,21,23,24,25,30,46,47], it should be noted that the entire process has been deeply influenced by the research carried out in the analysis of greenhouse gas emissions throughout the life cycle of roads [29].

It is also important to note that the study is carried out on a real construction project, a maritime infrastructure that expands the service of the port. Therefore, all the work units analyzed have been part of the construction of this structure.

On the other hand, although this aspect is based on estimation, a maintenance plan was prepared according to the project conditions, thus giving the opportunity to obtain a global perspective of the GHG emission levels generated throughout the operational life of the infrastructure, 25 years. Adding the maintenance phase to the research allows the achievement of a more complete and differential study as there are few studies that take into account this phase in the calculation, despite it being in the engineering sector.

Element analysis is also one of the strengths of this study. In machinery, more than 200 items were analyzed in the work, and therefore, a detailed search of the specifications of each machine in the catalogs formalized by the major brands was carried out. It can be established that approximately 90% of the data processed in this element have been accurately referenced, estimating only the performance of the remaining 10%. Different models were analyzed, fitted to the characteristics established in the work units, thus giving the possibility of obtaining the average performance of each machine. Regarding materials, the database of the most influential reference in this research was used. It should also be noted that a considerable percentage of these have been updated, thus adapting them to the project conditions.

The importance of this research is especially shown in the interpretation of the results [39]. The nonexistence of studies that analyze the GHG emissions generated in maritime constructions make this research clarify its results in comparison to others of a diverse nature.

According to the overall results, no clear differences were observed with other projects [29,48] as the distribution is approximately the same (80% machinery—20% materials). However, this ratio in the maintenance phase is less. Clearly, having fully created a maintenance plan through estimates, it has concentrated the distribution.

The extensive bibliographical search that was carried out made possible the creation of an extensive database, which proved essential for the calculation stage. The distribution of the results, both in the construction stage and in the maintenance stage, continue the previous trend established in other research, where the highest proportion of emissions is due to the off-road machinery, approximately 85%. These results are similar to those of other studies of different infrastructure projects [14].

Finally, the variety of scenarios reproduced shows the power and the capacity for improvement of current construction projects. As previously indicated, as machinery is the aspect with the greatest weight in this study, the most influential technical characteristics in the result were detected, with fuel being the key factor. By improving the efficiency of biodiesel, more positive results can be obtained in the immediate future.

In scenario 1 and scenario 2, it can be seen how the most advanced technologies focus on reducing local emissions rather than global ones, as has been shown in similar studies [48].

However, it is interesting to highlight that the methodology according to the fuel developed in scenario 3 is considerably distanced from the base result, so, although fuel is an important aspect to consider, it is better and more accurate to develop the methodology that encompasses the technical characteristics. In scenario 4, in which another database was used, the results agree with the base result; however, its value is approximately double. Despite the fact that the base case methodology is more specific and detailed, the ICE database has a greater data record, which allows more material batches to be calculated, thus obtaining a more bulky result.

Finally, it is interesting to check the results obtained in past studies. It is worth highlighting that the results obtained in this study (base case) are very similar to those published in related literature.

## 8. Conclusions

A serious problem such as the high carbon footprint generated in construction projects must be visualized in order to determine and evaluate the consequences of the environmental impact produced. Emission levels have been exceeded each year, and only drastic reductions in this trend have been observed in a situation as strange as the COVID-19 pandemic, which has completely stopped the world. The implementation of procedures that contribute at least to the visualization of this phenomenon must be immediate. The LCA not only fulfills this function, but due to its adaptability, it also indicates the critical elements that make up the construction project and the maintenance activities.

Probably, the circumstance that caused the low presence of LCA procedures in the world of maritime construction is the peculiarity of these infrastructures. It should be noted that this research has not considered the impact on the natural systems. However, it is necessary to take into account the presence in this location of the Posidonia meadows, an endemic species with high percentages of CO_2_ absorption and that, due to this characteristic, among others, it is totally illegal to modify its area.

An inventory was developed where all the elements that make up this construction project were analyzed (maintenance works included). This strategy allowed the development of a pioneering work in the calculation of the carbon footprint in maritime infrastructure through LCA. Therefore, the most critical activities and elements of the system were identified, in addition to presenting new alternatives using the scenarios established in this research. The most characteristic results are shown below (Table 8):

The main objectives planned were totally reached. We now have an initial order of magnitude for this kind of infrastructure, but the research and number of case studies should be continued in the future for different port typologies and areas to obtain reliable ratios of GHG emissions. The initial ratio of GHG emissions in construction and maintenance is calculated, where construction stage is the most important and off-road machinery is the main element from an LCA approach, as similar studies have concluded in infrastructure projects (ports were greatly left out of these studies).

## Figures and Tables

**Table 1:** CO_2_ emissions trend over the years.

Year	CO_2_ (Parts per Million—PPM)	Growth (PPM)
1960	315	0.71
1970	325	1.13
1980	340	1.70
1990	350	1.16
2000	370	1.23
2010	390	2.43
2019	410	2.60

**Table 2:** Summary of Life Cycle Analysis (LCA) methodology variables in a port infrastructure.

Concept	Assignment in Selected Project
Location	Spain, Europe
Life Cycle of the Port	50 years
Product system	Construction and maintenance
System limits	Work units
Impact category	Climate change assessment
Main GHG	CO_2_, CH_4_, N_2_O
Functional unit	Quay
GHG Major Parties	Dredging, dock, superstructure, esplanade, pavements
GHG minority items	Drainage, facilities, waste management, and health and safety
Main GHG agents	Machinery, materials, and natural systems

**Table 3:** Inspections and action during the maintenance phase.

Maintenance Plan: Operations											
N	Operation	Area	Surface (m^2^)	Main Material	Thickness (m)	Volume (m^3^)	Frequency (years)	Estimated Improvement	Operation during Useful Life	Operations/Useful Life	Material Volume/Frequency
1	Pavement improvement	New Dock	36,232	Bituminous mixture	0.06	2174	6	100%	4	0.16	8696
2	Pavement improvement	Old dock	31,000	Concrete	0.2	6200	12	100%	2	0.08	12,400
3	Caissons improvement	New Dock	-	Concrete	-	31,658	10	3%	3	0.12	2849
4	Caissons improvement	Old dock	-	Concrete	-	150,000	10	3%	3	0.12	13500
5	Breakwater replacement	New Dock	-	Rockfill	-	5100	4	6%	6	0.24	1836

**Table 4:** Emissions of off-road machinery in construction stage.

Off-Road Machinery			
Main Activity	Kg CO_2_	Kg CH_4_	Kg N_2_O
1. Demolition	216,000	4.00	20
2. Dock	15,800,000	300	1000
3. Superstructure	2,550,000	60	300
4. Esplanade	72,500,000	2700	800
5. Flooring	5,520,000	60	300
6. Drainage	448,000	10	50
7. Installations	128,000	4	10
8. Waste management	759,000	20	100
Total results	97,900,000	3160	9780
Kg CO_2_e results	97,900,000	265,000	2,580,000
Kg CO_2_e total	98,423,000		

**Table 5:** Total CO_2_e results obtained in the construction phase.

Total Results Tm CO_2_e: Construction			
**Total Machinery**	**101,000**	**Tm CO_2_e**	**87%**
Off Road Machinery	101,000	Tm CO_2_e	99.85%
Transport Machinery	156	Tm CO_2_e	0.15%
**Total Materials**	**15,600**	**Tm CO_2_e**	**13%**
Preproduction	13,600	Tm CO_2_e	87.18%
Production	2000	Tm CO_2_e	12.82%
Total Construction Stage	116,000	Tm CO_2_e	100%

**Table 6:** Total CO_2_e results obtained in the maintenance stage.

Total Results Tm CO_2_e: Maintenance			
**Total Machinery**	**339**	**Tm CO_2_e**	**65%**
Off Road Machinery	339	Tm CO_2_e	99.92%
Transport Machinery	0.26	Tm CO_2_e	0.08%
**Total Materials**	**181**	**Tm CO_2_e**	**35%**
Preproduction	11.9	Tm CO_2_e	6.58%
Production	169	Tm CO_2_e	93.42%
Total Maintenance Stage	520	Tm CO_2_e	100%

**Table 7:** Summary of the results obtained in each scenario, with the % of variation in scenarios in relation to the base case.

Construction Stage				
Case	Machinery		Materials	
	Off Road	Transport	Preproduction	Production
Base case (Tm of CO_2_e)	101,000	156	13,600	2000
Scenario 1	0.00001%	0.00001%	-	-
Scenario 2	0.00001%	0.00001%	-	-
Scenario 3	−7%	−8%	-	-
Scenario 4	-	-	+216%	

**Table 8:** Main results of this study.

	Construction	Maintenance	Cost (€)	Surface (m^2^)	kg CO_2_e	kg CO_2_e/m^2^
	Tm CO_2_e					
Base Case	117,000	520	34,060,648.00 €	130,510	3.43	899
